# Feasibility Analysis of Using Channel State Information (CSI) Acquired from Wi-Fi Routers for Construction Worker Fall Detection

**DOI:** 10.3390/ijerph20064998

**Published:** 2023-03-12

**Authors:** Runhao Guo, Heng Li, Dongliang Han, Runze Liu

**Affiliations:** 1 Department of Building and Real Estate, The Hong Kong Polytechnic University, Hong Kong, China; 2 Department of Building Environment and Energy Engineering, The Hong Kong Polytechnic University, Hong Kong, China

**Keywords:** channel state information, fall detection, construction worker, construction safety, commercial Wi-Fi router

## Abstract

Accidental falls represent a major cause of fatal injuries for construction workers. Failure to seek medical attention after a fall can significantly increase the risk of death for construction workers. Wearable sensors, computer vision, and manual techniques are common modalities for detecting worker falls in the literature. However, they are severely constrained by issues such as cost, lighting, background, clutter, and privacy. To address the problems associated with the existing proposed methods, a new method has been conceived to identify construction worker falls by analyzing the CSI signals extracted from commercial Wi-Fi routers. In this research context, our study aimed to investigate the potential of using Channel State Information (CSI) to identify falls among construction workers. To achieve the aim of this study, CSI data corresponding to 360 sets of activities were collected from six construction workers on real construction sites. The results indicate that (1) the behavior of construction workers is highly correlated with the magnitude of CSI, even in real construction sites, and (2) the CSI-based method for identifying construction worker falls has an accuracy of 99% and can also accurately distinguish between falls and fall-like actions. The present study makes a significant contribution to the field by demonstrating the feasibility of utilizing low-cost Wi-Fi routers for the continuous monitoring of fall incidents among construction workers. To the best of our knowledge, this is the first investigation to address the issue of fall detection using commercial Wi-Fi devices in real-world construction environments. Considering the dynamic nature of construction sites, the new method developed in this study helps to detect falls at construction sites automatically and helps injured construction workers to seek medical attention on time.

## 1. Introduction

Construction workers are often subjected to physically demanding work, and the construction industry records a significant number of workplace injuries, making it the most perilous occupation in the United States [1]. Falls are a primary cause of injury for construction workers, accounting for 15–30% of occupational injuries [2,3,4]. Occupational injuries that are similar in nature can have numerous adverse effects on construction projects, including schedule delays, increased project costs, and significant worker compensation claims [5,6,7,8]. The effectiveness of body treatment following a fall often depends on promptly detecting the fall and seeking medical assistance [9]. Delaying a worker’s access to medical attention after a fall can significantly increase the risk of death and lead to serious project accidents [10]. Identifying workers who have fallen and providing prompt assistance in case of a fall are crucial for mitigating project risks and ensuring the safety of construction workers.

Previous studies aiming to mitigate the hazards associated with fall accidents mainly focus on (1) using accident reports to analyze and avoid the main causes of falls [11,12,13] or relying on safety training and safety managers [14,15]; this approach is highly subjective and time-consuming, and it is difficult for the safety manager to cover the entire construction site comprehensively. (2) Automatic fall detection methods based on wearable devices [16,17,18,19,20,21]; these systems are only effective if users wear appropriate body sensors, which can negatively impact the work efficiency of construction workers. The additional weight and discomfort of the equipment may be unsuitable for use on hazardous construction sites and can also raise concerns related to health risks, high costs, and personal privacy. (3) Vision-based fall detection [22,23], which involves using cameras to monitor worker activity and identify falls. This technology raises concerns related to privacy, as the use of cameras can compromise it. In addition, the cost of installation is high, and the cameras can only detect falls from specific angles and are susceptible to interference from light [24,25].

**Figure 1:**
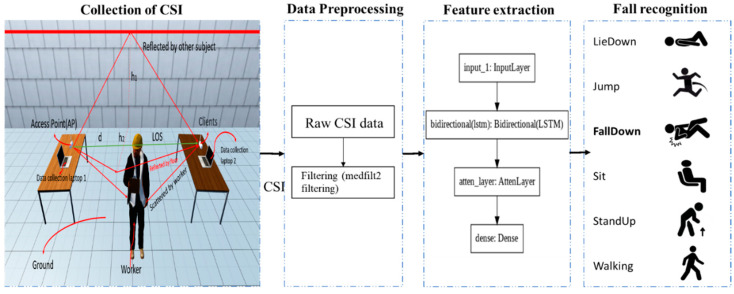
The flowchart for implementing worker fall recognition using CSI.

Given the limitations of previous studies, there is an increasing demand for automated, non-invasive, and continuous monitoring methods that can effectively monitor falls among construction workers. The Wi-Fi-based approach is an emerging solution for fall recognition that addresses the above limitations [26]. Channel State Information, also known as CSI, is how the Wi-Fi signal is emitted from the transmitter and is attenuated by a series of refractions and reflections to the receiver [27]. The CSI changes as a result of body part movements, altering how the wireless signal is reflected [28]. By monitoring CSI data streams that correspond to various activities and comparing them with a trained model, it is possible to identify human movements. Fall detection can be achieved by extracting features from the CSI data streams and applying appropriate machine-learning techniques for model training. Commercial Wi-Fi routers are widely used in our daily lives [26]. Compared to other behavior recognition methods, Wi-Fi-based activity recognition is low-cost and easy to deploy without purchasing additional dedicated equipment. The three primary types of Wi-Fi signals used to identify activities are received signal strength indicators (RSSI), signals based on specialized radio hardware, and channel status information (CSI) [29]. It is challenging to recognize human activity at a finer scale using RSSI due to its limited sensing capacity and low signal resolution. Dedicated radio hardware is also costly to set up because it is not readily accessible. Therefore, this study proposes using CSI signals to recognize construction workers’ fall accidents. In the context of this research, this study aims to investigate the feasibility of using CSI for worker fall monitoring at construction sites, thereby enabling a new research direction for construction safety. The flowchart for implementing worker fall recognition using CSI is shown in Figure 1. The initial step involves gathering Channel State Information (CSI) data with a laptop and Wi-Fi router, followed by CSI pre-processing, and finally, a classification model is used to identify the CSI data.

This paper is structured as follows: Section 2 provides a comprehensive review of the related literature in the field. The methodology employed in the study is thoroughly discussed in Section 3. The process of collecting CSI data and the results of the recognition experiment are described in Section 4 and Section 5, respectively. The findings are then analyzed and discussed in Section 6, and a conclusion is drawn in Section 7.

## 2. Background

Falls on construction sites are a common cause of accidents for workers [30], which are sometimes fatal. Accidental falls are a serious and ongoing issue in many nations [31,32,33]. Fall hazards result from prolonged exposure to fall hazards that arise from environmental or perceived factors [34]. A lack of concentration and physical fatigue due to overwork without adequate rest are the main reasons why construction workers are prone to falls at construction sites [34,35,36,37]. In addition, unsafe conditions such as slippery surfaces, uneven platforms, ground obstacles, and stairs, which are often found on construction sites, can also expose construction workers to the risk of falls [38,39]. The construction site is, therefore, a highly dangerous place, especially concerning the potential for falls.

### 2.1. Common Fall Detection Techniques Used on Construction Sites

#### 2.1.1. Manual-Based Fall Monitoring Methods

Over the years, construction companies have tried to use adequate safety training and the intervention of safety managers to reduce the likelihood of worker accidents [40,41]. However, this traditional manual approach is inadequate to deal with the various fall hazards in today’s dynamic working environment. As the construction work environment is constantly changing and not easily controlled, it is impractical to rely solely on the safety manager to continuously and remotely monitor all worker risks throughout the workplace [38,41,42]. Workers are still exposed to environments with a high risk of falls. In order to cope with the uncertainty of the modern construction environment, advanced technological aids are needed to improve the timeliness and continuity of fall detection.

#### 2.1.2. Wearable-Device-Based Fall Monitoring

Recently, many studies have demonstrated the effectiveness of wearable technology for worker fall monitoring. Wearable technology can collect a large amount of data from recognized physical responses and thus be used to monitor physical changes while working in the workplace. These devices include accelerometers, gyroscopes, and other technologies that provide solutions for measuring falls [19,43,44]. Typically, measuring the loss of balance (LOB) is the primary method of monitoring worker falls using wearable sensors [45]. When a worker is in danger of falling, the worker will develop an abnormal gait pattern, which can be used to detect falls [46]. A large number of studies have therefore used IMU sensors attached to the waist or other body parts to measure the LOB caused by fall hazards [19,44,45,47,48,49].

Similarly, wearable insole pressure systems are used to measure plantar pressure and ground reaction forces to extract information for fall detection in construction workers [50]. Only if all sensors are worn or carried by the worker at the moment of the fall accident will these fall detection systems work. It is challenging for construction workers to comply with the rule that sensors must always be worn on the body. The extra burden of equipment is particularly uncomfortable for construction workers involved in complex labor tasks for long periods.

#### 2.1.3. Vision-Based Fall Monitoring

Computer-vision-based fall detection relies on cameras mounted at specific locations to capture images or video sequences for activity recognition and classification. The fall activity is separated from other events using an activity classification algorithm. The latest research based on infrared and depth cameras has expanded their range of applications and has demonstrated their high recognition accuracy [24,25,51]. However, these methods are significantly hampered by several challenges, including high installation costs, dependence on adequate lighting, invisibility, and privacy concerns [24,25].

### 2.2. CSI-Based Fall Monitoring for Construction Workers

#### 2.2.1. Basic Theory

Activity recognition technology using wireless signals is a non-intrusive and privacy-protective method [52,53]. Based on the IEEE 802.11 series, ordinary home wireless devices are commonplace. The cost of using Wi-Fi signals is very low, as Wi-Fi signals are universally available. With the development and commercialization of Wi-Fi technology, CSI-related research can be carried out using common commercial Wi-Fi equipment, which significantly reduces the cost of research [54,55,56].

The transmission of a signal by a transmitter undergoes a series of attenuations and reflections before it reaches the receiver, and this variation is referred to as the Channel State Information (CSI). CSI captures the path of a wireless signal at a specific carrier frequency from the transmitting antenna to the receiving antenna. The basis of CSI-based activity recognition lies in the fact that the amplitude and phase of the CSI signal will deviate from their normal measurements when an object or a person moves between the transmitter and the receiver antenna, thereby inducing movement-related perturbations.

#### 2.2.2. The Relationship between CSI and Worker Falls

**Figure 2:**
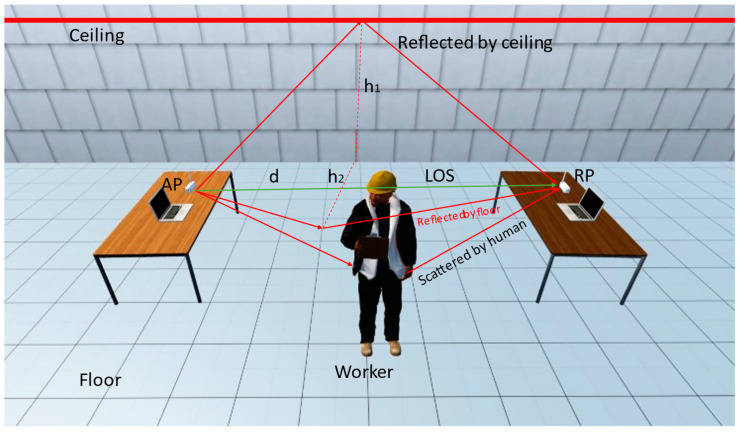
The wireless propagation model.

We can measure the value of CSI propagated by the transmitter and receiver for behavior recognition purposes. As shown in Figure 2, the wireless propagation model is established based on the path attenuation model [52]. Wi-Fi signals are propagated through physical space by reflections from various objects. The propagation of wireless signals is limited by physical space, resulting in the signals received carrying information regarding the routes through which they have traveled. If construction workers are present in the environment, scattering from the human body introduces additional signal paths [53]. CSI must therefore have some connection to worker falls.

The formula of the overall propagation model is as follows:

$$P_{r}\left(d\right)=\frac{P_{t}G_{t}G_{r}\lambda^{2}}{{\left(4\pi \right)}^{2}{\left(d+4h+\Delta \right)}^{2}}$$

where $P_{t}$ represents the transmit power; $P_{r}\left(d\right)$ is the received power; $G_{t}$ and $G_{r}$ are the amount of transmit gain and the amount of receive gain, respectively; λ indicates the wavelength; *d* and *h* are the propagation distance and indoor space height, respectively; and Δ denotes the approximate amount of signal disturbance caused by the presence of personnel.

CSI describes the quality change in the environmental reflection experienced by the signal during the propagation process and is based on the evaluation matrix of the signal propagation quality at the signal-receiving end in Orthogonal Frequency Division Multiplexing (OFDM) technology [57]. The physical layer of current commercial wireless devices uses OFDM, complies with IEEE 802.11 n/ac standards, and allows Multi-Input Multi-Output (MIMO) technology to communicate. This advantage provides sufficient conditions for extracting adequate CSI information [56].

### 2.3. Knowledge Gaps and Research Objectives

As described in Section 2.1, previous research was based on manual detection, wearable devices, computer vision, and other techniques to detect worker falls on construction sites. However, they have obvious limitations. To overcome the limitations of these studies, this study proposes using CSI data extracted from Wi-Fi devices for fall monitoring of workers at construction sites. The first advantage of using CSI is its objectivity and time-saving nature. Unlike manual detection, which requires the safety manager to always focus on all workers, CSI-based methods can detect falls automatically and provide immediate alerts. This approach can save time and reduce the risk of human error in detecting falls. Another advantage of using CSI is its non-invasive nature. Unlike wearable sensors, which can be uncomfortable and require individuals to wear them, CSI uses existing Wi-Fi infrastructure, which does not interfere with individuals’ daily activities. Additionally, CSI does not require line of sight or complex camera setups, making it a low-cost and easy-to-deploy approach for fall detection. CSI also provides privacy advantages, as it does not capture images or personal data. This is especially important for workers who may be reluctant to wear sensors or cameras due to privacy concerns. By using CSI, privacy can be maintained, and individuals can be assured that their personal information is not being recorded. However, one of the main disadvantages of using CSI is that its accuracy can be affected by various factors, such as the location of the Wi-Fi router and interference from other moving objects. In addition, CSI is susceptible to interference from other wireless transmitter devices on the same frequency, which may also affect Wi-Fi signal variations. Despite these limitations, CSI-based fall detection remains a promising approach to preventing falls in the workplace and improving the safety of workers. Although the use of CSI signals to detect falls is a promising strategy, no study has analyzed and experimented with its feasibility. In this context, this paper investigates the feasibility of using CSI to detect falls among construction workers. Specifically, this study demonstrates that CSI signals can accurately identify construction worker falls on actual construction sites, which helps provide timely access to medical care for injured construction workers.

## 3. Methodology

### 3.1. Data Collection Method

The collection of CSI data is a crucial step in the process of fall detection in construction sites. Channel State Information (CSI) is a wireless communication concept that pertains to information about the characteristics of the wireless channel between a transmitter and a receiver. It encompasses crucial information, including the signal’s amplitude, phase, and frequency response, which can be utilized to extract various signal features. CSI is particularly sensitive to movement and location changes of objects and people in the environment. The foundation of CSI-based activity recognition is grounded in the observation that the amplitude and phase of the CSI signal will undergo alterations from their typical measurements when an object or person moves between the transmitter and receiver antenna. This causes movement-related perturbations, which can be detected and utilized to recognize various activities.

The widespread usage of commercial routers in these environments makes them an attractive choice for data collection. Two popular tools used for CSI data acquisition are the Linux 802.11n CSI Tool and the Atheros CSI Tool. The Linux 802.11n CSI Tool, although effective, requires outdated hardware and often necessitates modifications to the computer being used. As a result, the Atheros CSI Tool is preferred, as it is an open-source tool that enables the extraction of wireless communication data from Wi-Fi network cards. This information includes CSI, the received packet payload, and other relevant details, such as the Timestamp, RSSI per antenna, and Data Rate.

The receivers and transmitters are two Wi-Fi routers with built-in Atheros AR9580 NICs equipped with OpenWrt systems. In this study, the injector monitor mode was used to send and receive CSI data. The monitor mode allows the modulation of the transmission rate, the number of packets sent, the number of transmitting antennae, and the short/long guard interval. It is essential to invoke the monitor mode, which allows for more standard CSI data to be obtained for subsequent model training. Two laptops with Intel Core i5 series processors, Windows 10, Xshell6, and Winscp were selected as the processing terminals to extract the received CSI data.

**Figure 3:**
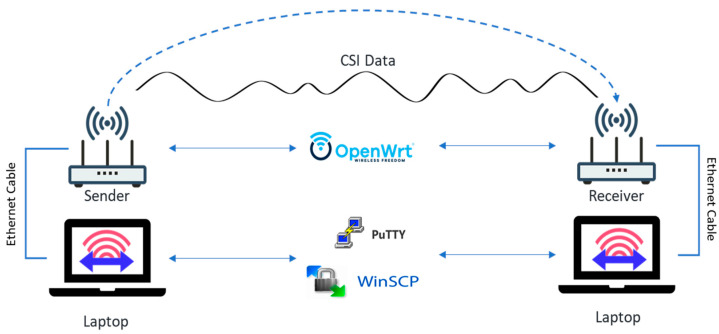
Data collection equipment.

This study represents a significant advancement in prior research through the design of a system that incorporates Tx-Rx communication to calculate the Channel State Information (CSI) and collect data for analysis. The system operates by having the transmitter continuously transmit Wi-Fi packets while the receiver receives the data and computes the CSI. In order to address the limitation of the router’s limited data storage capacity, the system has been optimized through the following measures: (1) the transmitter transmits data to the receiver, which then calculates the CSI; (2) the raw CSI data are extracted from the receiver by the user’s laptop and processed, saved, and visualized using MATLAB R2021a. The procedure for obtaining CSI data is illustrated in Figure 3.

### 3.2. CSI Data Pre-Processing Method

**Figure 4:**
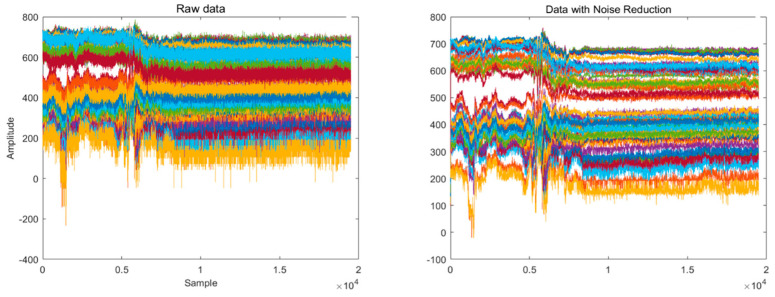
The amplitude of CSI data before and after noise reduction (Each set of CSI data contains 56 subcarriers. Each subcarrier is represented by a color).

CSI data consist of two components: phase and amplitude. During the subsequent data analysis in this paper, we use only amplitude information for fall identification. As the transmit power and rate vary, unpredictable impulsive noise is generated between the transmitter and receiver. The raw CSI data received have a lot of noisy information that is irrelevant to the worker’s actions. To avoid losing any possible data information, the pre-processing of CSI data in this paper is limited to the removal of obvious noise. In this study, a median filter was introduced for noise reduction. The change in the amplitude of CSI data before and after noise reduction is shown in Figure 4 below.

### 3.3. Fall Detection Method

Previous studies have investigated the application of various machine-learning algorithms for fall recognition, including deep recurrent neural networks (RNNs), support vector machines (SVMs), and logistic regression algorithms. The selection of a particular algorithm is based on its ability to analyze and classify the collected CSI data, leading to accurate fall detection. The action recognition process is akin to speech recognition. The utilization of Long Short-Term Memory (LSTM) can mitigate the potential for gradient disappearance or explosion during neural network training and result in high recognition accuracy [58].

Fall behavior often exhibits both pre-fall features, such as body tilt, and post-fall features, such as body smoothness. To consider both past and future information, bidirectional LSTM (BLSTM) is used for activity recognition in this paper. BLSTM provides a comprehensive understanding of both pre-fall and post-fall features. The use of LSTM offers two advantages. Firstly, LSTM is capable of automatically extracting features, obviating the need for pre-processing data, which meets our expectations. Secondly, LSTM preserves the temporal state information of the activity, enabling differentiation between similar activities such as “lie down” and “fall”. Although these activities exhibit similar velocity characteristics, they have distinctive pre-states and post-states. The memory capacity of LSTM allows for the identification of these activities.

## 4. Experiments

### 4.1. Experimental Design

The experiment was carried out using two Wi-Fi routers with Atheros 9580 NIC chips and two laptops. One laptop served as a transmitter, and the other acted as a receiver; both were connected to a single network. Data are sent and received through a local area network between the transmitter and receiver before being sent to the computer performing the processing. The transmission rate must be high enough (around 1 kHz) to record the instant of a recently completed fall for the CSI to demonstrate substantial changes brought on by movement. This is because when the sampling rate is around 50 Hz, we observe serious degradation in the performance of the classification method, even though it runs much faster. The cost of denoising and feature extraction increases when the sampling rate is increased because more samples are taken. Accurate recognition may not be improved by increasing the sample rate. Therefore, a good compromise between the computational cost and accuracy can be obtained by choosing a suitable sampling rate (approx. 1 kHz). In total, 1000 Wi-Fi packets are delivered every second, and the Atheros CSI Tool extracts the CSI from the Wi-Fi signal. In the experiment, two receiving antennae and one broadcasting antenna were set up, making a total of two communication lines. Personnel operation information was collected at a 2.4 GHz center frequency.

**Figure 5:**
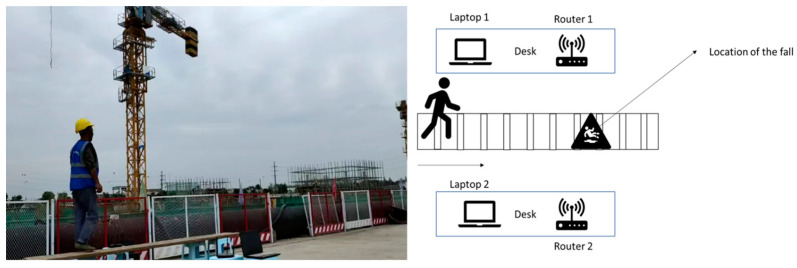
Experimental scenario.

To collect the most realistic experimental data on the fall behavior of construction workers, the experiment was set up on an actual construction site. The distance between the transmitter and receiver was set at 4.0 m, and the height was set at 0.5 m for line-of-sight circumstances (this was set to obtain a larger signal-to-noise ratio for sensing human movement). The transmitter and receiver were positioned 1 m above the ground to provide a clear signal path. The perceived real-life experimental scenario and its planar structure are shown in Figure 5.

### 4.2. Description of the Dataset

Six construction workers who were present at the site provided the CSI data for this experiment (two carpenters and four electricians). Traditional CSI-based behavior recognition typically focuses on undisturbed laboratory environments, but actual construction sites are subject to numerous disruptive factors, such as construction noise, vibrations, and irregular building walls, which can affect the propagation of CSI. To demonstrate the experiment’s rigor, the test site was located in a wastewater treatment facility renovation project in Chengdu, Sichuan Province, which included an operating tower crane and excavator. Collecting data in this way realistically captures CSI fluctuations due to background factors. Data collection began on 4 September 2022. Four men and two women, ages 20 to 50, with five to sixteen years of work experience in the construction industry, made up the participants. None of the individuals reported having any clinical issues that would limit their capacity for everyday activities on a physical or mental level. The six construction workers’ demographic details and the number of groups for which data were gathered are shown in Table 1.

The various characteristics of different individuals can increase the variety of training samples and make it easier to identify various worker falls using artificial neural networks. There may be differences in the detection rates among workers based on their height, weight, and gender. The accuracy of CSI-based fall detection depends on the signal strength and quality of the received Wi-Fi signals, which may be affected by the worker’s body composition and orientation. For instance, taller or heavier workers may cause more signal attenuation due to the increased number of obstacles and absorption of the Wi-Fi signal. Similarly, the orientation of the worker’s body may also affect the signal. Gender may also play a role in the accuracy of fall detection since men and women tend to have different body compositions and postures, leading to differences in signal attenuation and variations in the received Wi-Fi signal.

**Figure 6:**
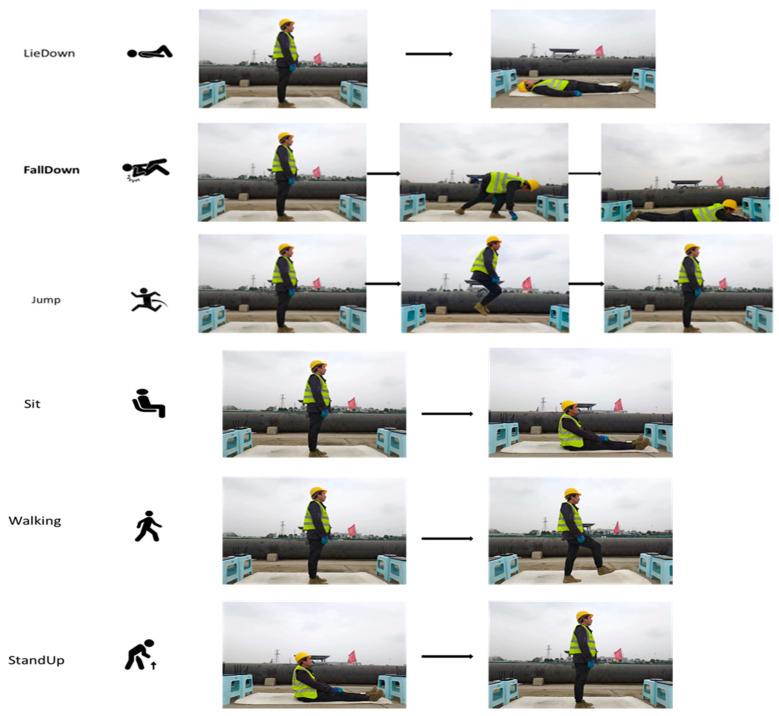
Details of six activities.

These factors can be mitigated by increasing the sample size. For example, collecting data from more workers can reduce the impact of individual worker characteristics, such as height, weight, and gender, on the accuracy of the test. This approach can help in obtaining more reliable results and enhancing the generalizability of the findings. There is no clear rule for how many samples are required to train a neural network; instead, it is an iterative process. The quantity of data required for the dataset depends on the difficulty of the activity and the technique used. A total of 60 sets of experiments were conducted by each of the six construction employees who were given the task of participating in the study. These activities were “LieDown”, “FallDown”, “Jump”, “Sit”, “StandUp”, and “Walking”. The details of these activities can be seen in Figure 6.

**Figure 7:**
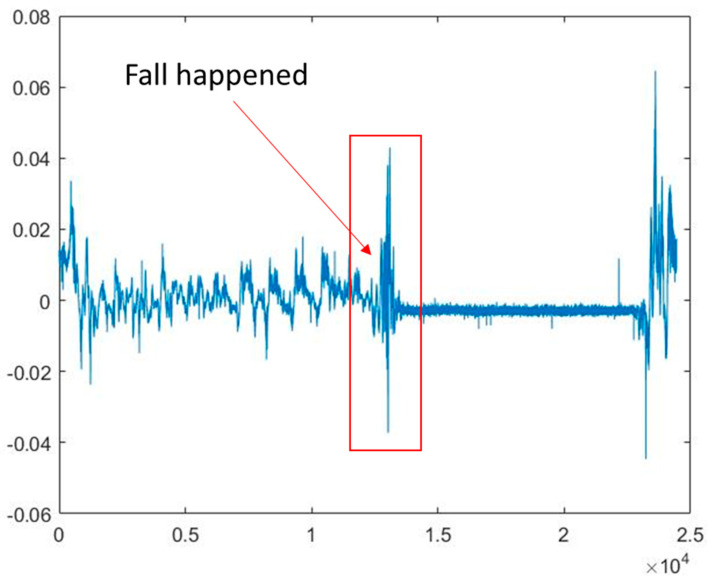
Correspondence between fall and CSI signal amplitude.

CSI data for actions other than falls were collected to demonstrate that a CSI-based fall recognition model can accurately distinguish between falls and fall-like behaviors. Only predefined actions were executed at intermediate intervals during the entire data acquisition process. More specifically, the worker was virtually steady at the start and finish of the data collection. The experiment was manually run, so there was always a chance that the times it took to start and end would vary slightly. Each worker performed each action ten times, with the duration of individual tasks set at approximately 15 to 25 s. The overall image was recorded at the same time as the data were collected, thus ensuring that the timestamps correspond to the data annotation at a later stage. A total of 360 data streams (6 worker activities, 10 repeated trials, and 6 participants) were gathered for this study. Each action dataset contains 56 subcarriers, each containing approximately 20,000 samples. The relationship corresponding to the fall occurrence and the change in CSI signal amplitude is shown in Figure 7 below. The fall behavior occurred during sharp fluctuations in CSI amplitude.

**Figure 8:**
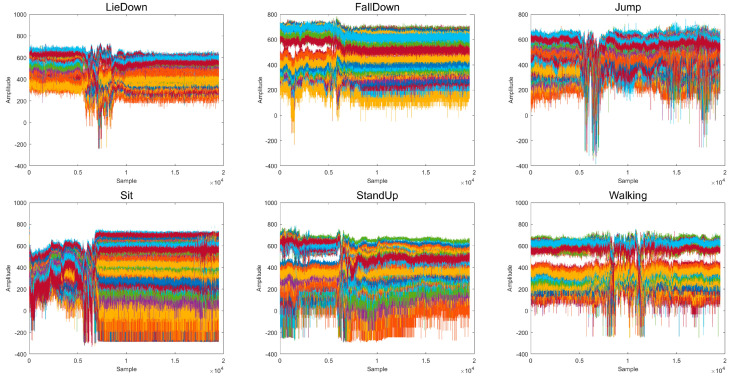
Correspondence between different activities and CSI signal amplitude (Each set of CSI data contains 56 subcarriers. Each subcarrier is represented by a color).

The CSI amplitudes of the 56 subcarriers collected for the 6 actions as a function of time are shown in Figure 8 below. It can be seen that the different worker actions have significantly different effects on the actual CSI signal.

## 5. Results

The LSTM technique differs from traditional approaches in that it can directly extract CSI features without using PCA or STFT. The network receives 56 × 19,000 (feature samples) features as its input and outputs a probability for each of the six classes. Two hundred hidden units are picked, with just one hidden layer being taken into consideration. We utilized Stochastic Gradient Descent (SGD) for the numerical optimization of cross-entropy with a batch size of 200 and a learning rate of 1 × 10^−4^. One disadvantage is the lengthy training period associated with employing LSTM in this manner. However, one may also leverage GPUs and accelerate training by utilizing deep learning tools such as TensorFlow. The test may be completed quickly after LSTM has been trained. To assess the effectiveness of the classification model, we measured the precision, loss rate, and accuracy.

**Figure 9:**
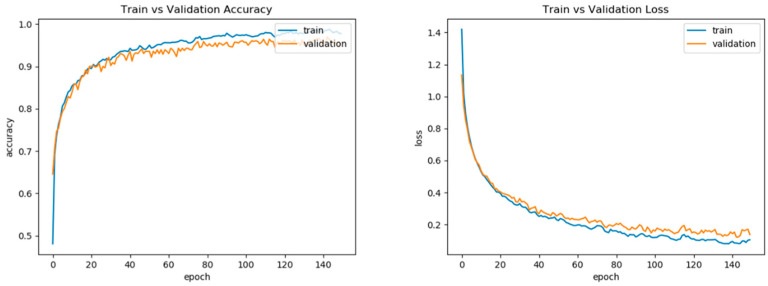
The accuracy (**Left**) and loss (**Right**) of the trained model based on bidirectional LSTM.

Figure 9 provides an illustration of the accuracy and loss of the proposed fall detection system’s training process. We can observe that, during training, the network’s performance stabilizes after around 100 iterations.

**Figure 10:**
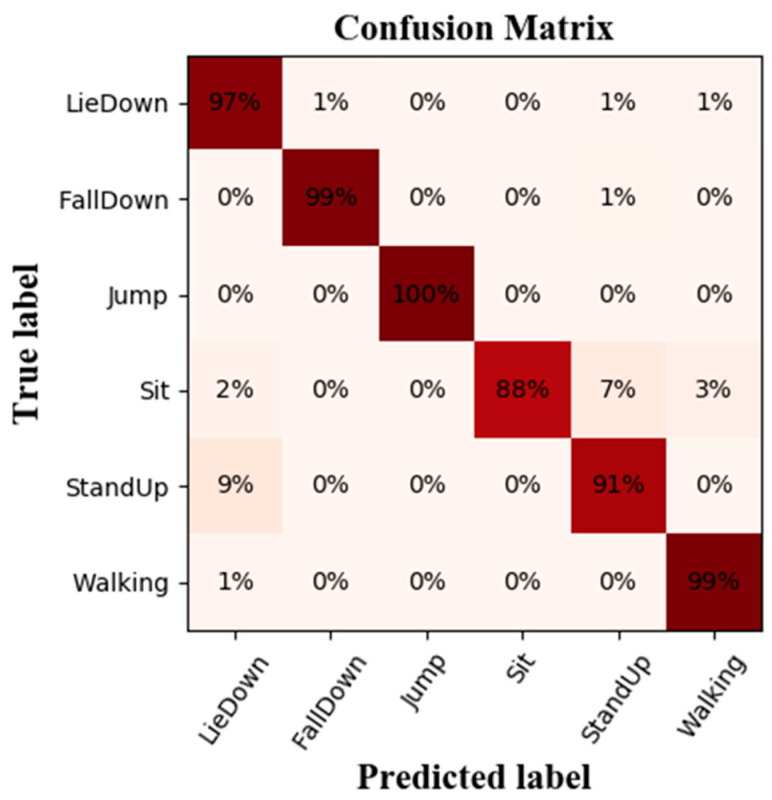
The confusion matrix of the model.

The CSI values for various activities vary, which affects the recognition accuracy. For each activity, we use a confusion matrix (also known as an error matrix) to indicate how well our suggested classifier performed. The rows correspond to the predicted categories, while the columns correspond to the actual classes. The results in the confusion matrix (Figure 10) show an accuracy of over 88% for all of these activities. For “LieDown”, “FallDown”, “Jump”, “Sit”, “StandUp”, and “Walking”, the mean detection rates for the six movements were 97%, 99%, 100%, 88%, 91%, and 99%, respectively.

Table 2 illustrates the fall detection accuracy of the CSI-based fall detection system proposed in this study in comparison to fall detection systems based on computer vision and wearable sensors. The table reveals that the CSI-based fall detection technology is capable of meeting practical usage requirements in terms of fall detection accuracy.

Activities that involve more prominent limb movements, such as “FallDown”, “Jump”, and “Walking”, exhibit higher recognition accuracy (as shown in Figure 10) due to their significant impact on CSI features. In addition, the recognition rate of fall activity is of the utmost importance for fall detection. The BLSTM networks we trained were 99% accurate for fall recognition, which makes these models very effective for fall detection systems on construction sites. Another observation is the relatively low accuracy of recognition of the actions “Sit” and “StandUp”. One possible explanation for this phenomenon is that these activities result in similar changes in CSI values, as they involve similar movement speeds. Nonetheless, the recognition accuracy is considered acceptable, and the problem could potentially be resolved in the future by expanding the dataset.

## 6. Discussion and Future Work

### 6.1. Performance of CSI-Based Fall Detection Method

This paper presents a low-cost Wi-Fi-based fall detection method for construction workers. The approach employs wireless routing to acquire a CSI signal, which is subsequently utilized to extract features and identify construction worker falls. The findings of this study demonstrate that worker behavior can impact the CSI signal, and these interferences can be measured using the proposed LSTM. The study investigates six different types of common behaviors on construction sites. The results show a robust correlation between worker behavior and the amplitude of the CSI signal. These observations indicate that the proposed method can effectively identify the fall events of construction workers with a high fall detection accuracy.

The use of CSI data extracted from Wi-Fi devices for fall monitoring of workers at construction sites can overcome the limitations of previous research. Unlike manual detection, which demands continuous attention, CSI-based methods mitigate the likelihood of human error. In addition, CSI technology does not disrupt daily operations and does not necessitate line-of-sight or intricate arrangements. Furthermore, CSI-based methods do not capture images or collect personal data, making them particularly valuable for individuals who have privacy concerns.

### 6.2. Limitations

This study demonstrates the potential of the suggested method for detecting falls of construction workers in controlled experimental settings, but there are still some limitations to consider. Firstly, the conducted experiment only tested activity without any additional tasks, such as carrying materials. Carrying objects can alter the body’s center of gravity and modify the pattern of radio waves reflected by the body, potentially impacting the accuracy of the CSI-based fall detection system. Therefore, it is necessary to investigate the impact of carrying objects and other disruptions on CSI in real-world settings to demonstrate the feasibility of the proposed approach. Moreover, the study needs to explore how diverse characteristics of construction workers, including work experience, age, and gender, affect CSI.

Secondly, to ensure the safety of construction workers, the experimental scenarios were conducted on relatively flat construction site roads. This led to a lack of specific data on falls in more complex construction scenarios, such as falls in workers climbing ladders or walking on high-altitude scaffolding. Furthermore, the presence of other moving objects around workers, such as construction equipment, can result in significant changes in the CSI amplitude and phase. Future research should focus on improving the model’s robustness to function effectively in complex scenarios involving multiple moving objects. Although the preliminary results are promising, the system described is still in its early stages, and more complex scenarios should be investigated and analyzed to enhance the decision module of the fusion system.

Finally, to implement the CSI-based fall detection system in practical construction site scenarios, the limitations of manual data transmission and model training need to be addressed to reduce the cost of human technological resources. Future research needs to extend this study to develop a fully automated system for recognizing worker fall activities, which includes creating an operation-friendly visual monitoring interface and embedded systems. Such investigations could provide valuable insights into the limitations and potential of using CSI for fall detection in real-world scenarios.

### 6.3. Future Directions

Several outstanding challenges require further attention in future work, including utilizing the phase information of CSI in addition to the magnitude, determining the optimal placement location for the equipment, and identifying the behavior of multiple workers simultaneously.

#### 6.3.1. CSI Phase Information

The underutilization of phase information derived from Channel State Information (CSI) for activity recognition in the literature can be attributed to the presence of errors, including Carrier Frequency Offset (CFO) and Sampling Frequency Offset (SFO). Nevertheless, these errors can be effectively mitigated by subtracting the phase information from neighboring antennae, resulting in the calculation of the phase difference. The phase difference is correlated with the Angle of Arrival (AOA); any variations in the target’s position lead to corresponding fluctuations in the AOA and, subsequently, the phase difference. The rapid and substantial motion of the target scatters the signal in a more randomized manner, leading to a more dynamic and rapid change in the AOA and phase difference. Utilizing these variations in phase difference and amplitude as features for activity classification through the implementation of a suitable machine-learning algorithm remains an area for future investigation.

#### 6.3.2. Wi-Fi Router Placement in Construction Sites

The focus of this work is on the use of CSI-based methods for fall detection on construction sites. However, finding the best location to install a Wi-Fi router to ensure good signal coverage and excellent detection performance is not easy. In the future, we plan to investigate the best locations to place the routers to optimize signal coverage and fall detection.

#### 6.3.3. Multi-Worker Activity Recognition

The present study focused on the recognition of falls in a single construction worker. However, recognizing falls among multiple workers simultaneously presents a more complex and intriguing challenge. The deployment of multiple receivers may offer an added advantage in accurately differentiating between the activities of multiple individuals. Further investigation in this direction is an area for future study.

## 7. Conclusions

This study investigated the potential of using CSI data from commercial Wi-Fi routers to monitor construction workers’ falls at construction sites. To achieve this, data were collected from six construction workers in a fall experiment with 360 sets of activities. The results showed that (1) there was a significant correlation between changes in the CSI amplitude and construction workers’ fall behavior and (2) CSI signals extracted from commercial routers could accurately discriminate between falls and fall-like movements, identifying fall movements with 99% accuracy. The main contribution of this study is to demonstrate the feasibility of using commercial Wi-Fi routers for construction worker fall monitoring. This research is the first attempt to use the new CSI data for the fall identification of construction workers on construction sites. The objective and continuous fall monitoring of construction workers, in a non-invasive manner, is important due to the complexity and dynamic nature of their behavior. This study will enable timely access to medical care for injured workers and improve the overall safety of the construction site.

This study also suggests future research directions for using CSI data for fall detection on construction sites. One area is utilizing the phase information of CSI in addition to the magnitude of activity recognition. The study also highlights the need to determine the optimal placement location for Wi-Fi routers to ensure good signal coverage and excellent detection performance. Simultaneously recognizing falls among multiple workers is another area for future investigation, which may involve deploying multiple receivers.

## Figures and Tables

**Table 1:** The demographic information for participants in the experiment.

Worker No.	Age (Years)	Height (cm)	Weight (kg)	Working Experience (Years)	Occupation	Gender	Number of Experiments
1	33	185	79	5	Carpenter	Male	60
2	32	183	80	9	Carpenter	Male	60
3	29	175	75	6	Electrician	Male	60
4	40	165	63	15	Electrician	Male	60
5	41	169	65	16	Electrician	Female	60
6	38	172	69	9	Electrician	Female	60

**Table 2:** Fall detection accuracy of fall detection systems using different methods.

Methods	Accuracy	References
Computer Vision	98.0%	[59]
	99.0%	[60]
Wearable Sensor	97.6%	[61]
	94.1%	[62]
Channel State Information (CSI)	99.0%	

## Data Availability

The data presented in this study are available on request from the corresponding author. The datasets generated and/or analyzed during the current study are not publicly available due to ethical restrictions.
